# Occupation Dynamics and Impacts of Damselfish Territoriality on Recovering Populations of the Threatened Staghorn Coral, *Acropora cervicornis*

**DOI:** 10.1371/journal.pone.0141302

**Published:** 2015-11-18

**Authors:** Stephanie A. Schopmeyer, Diego Lirman

**Affiliations:** Department of Marine Biology and Ecology, Rosenstiel School of Marine and Atmospheric Science, University of Miami, Miami, Florida, United States of America; National University of Singapore, SINGAPORE

## Abstract

Large-scale coral reef restoration is needed to help recover structure and function of degraded coral reef ecosystems and mitigate continued coral declines. *In situ* coral propagation and reef restoration efforts have scaled up significantly in past decades, particularly for the threatened Caribbean staghorn coral, *Acropora cervicornis*, but little is known about the role that native competitors and predators, such as farming damselfishes, have on the success of restoration. Steep declines in *A*. *cervicornis* abundance may have concentrated the negative impacts of damselfish algal farming on a much lower number of coral prey/colonies, thus creating a significant threat to the persistence and recovery of depleted coral populations. This is the first study to document the prevalence of resident damselfishes and negative effects of algal lawns on *A*. *cervicornis* along the Florida Reef Tract (FRT). Impacts of damselfish lawns on *A*. *cervicornis* colonies were more prevalent (21.6% of colonies) than those of other sources of mortality (i.e., disease (1.6%), algal/sponge overgrowth (5.6%), and corallivore predation (7.9%)), and damselfish activities caused the highest levels of tissue mortality (34.6%) among all coral stressors evaluated. The probability of damselfish occupation increased as coral colony size and complexity increased and coral growth rates were significantly lower in colonies with damselfish lawns (15.4 vs. 29.6 cm per year). Reduced growth and mortality of existing *A*. *cervicornis* populations may have a significant effect on population dynamics by potentially reducing important genetic diversity and the reproductive potential of depleted populations. On a positive note, however, the presence of resident damselfishes decreased predation by other corallivores, such as *Coralliophila* and *Hermodice*, and may offset some negative impacts caused by algal farming. While most negative impacts of damselfishes identified in this study affected large individual colonies and <50% of the *A*. *cervicornis* population along the FRT, the remaining wild staghorn population, along with the rapidly increasing restored populations, continue to fulfill important functional roles on coral reefs by providing essential habitat and refuge to other reef organisms. Although the effects of damselfish predation are, and will continue to be, pervasive, successful restoration efforts and strategic coral transplantation designs may help overcome damselfish damage by rapidly increasing *A*. *cervicornis* cover and abundance while also providing important information to educate future conservation and management decisions.

## Introduction

Decades of drastic decline have reduced the once-dominant reef-building coral genus *Acropora* into a now minor component of shallow Caribbean reef communities and have prompted its listing as threatened under the US Endangered Species Act [1]. While large-scale propagation and restoration practices are gaining popularity to restore ecosystem structure and function to degraded coral reefs, the recovery of severely depleted wild *Acropora* populations is commonly faced with numerous technical and ecological challenges. Therefore, restoration practitioners have recently developed science-based methodologies to mitigate further losses and aid in the recovery of this important coral genus [2,3]. Having overcome the initial technical difficulties associated with producing large numbers of coral ramets, *in situ* coral propagation and reef restoration efforts in Florida and the Caribbean have scaled up significantly in the past decade, with 10,000s of staghorn colonies (*Acropora cervicornis*) now routinely propagated in underwater coral nurseries [4]. However, the transplantation of nursery-grown corals onto wild reefs poses particular challenges as coral fragments and small coral colonies are placed in reef environments that look considerably different from the habitats where *Acropora* populations were dominant only 30–40 years ago. The demise of the long-spined sea urchin, *Diadema antillarum*, in the 1980s, overfishing, and the increased frequency of temperature anomalies, disease outbreaks, and coastal development have modified present reef environments so that recovering *Acropora* populations now face synergistic environmental challenges that are rarely controlled as part of restoration efforts and will likely determine whether these important reef components can regain their pre-eminent keystone role within coral reefs. In addition, coral reefs are already facing decreases in net calcification and increases in overall net erosion, in part due to ocean acidification [5,6], which will continue to degrade reef structure and will influence the potential for recovery of coral reefs. In this study, we explore the impact of common native territorial damselfishes, and their interactions with their preferred microhabitat, *A*. *cervicornis* [7–10]. Previous observations have shown that, when abundant, staghorn populations remain largely unaffected by the gardening activities of damselfish but that, after significant declines in staghorn abundance, the concentration of damage by damselfishes on a diminished prey can have negative effects on the long-term persistence of the coral population [11,12]. Here, we evaluate the threat that damselfish occupation and gardening activities pose to the condition and fate of wild and restored *A*. *cervicornis* populations in Florida.

The effect of predation on endangered species [13–15] and the potential for predation to inhibit the recovery of depleted species has been well documented [16]. In many cases, restoration failures have been linked directly to interactions between the restored species and exotic or introduced species [17,18]. To date, little is known about the role of native predators on the success of restored native species, especially within coral reefs [19]. Predation is known to be a potential source of significant coral mortality [20,21] and the relative impact of predation is likely to increase as coral cover (i.e., prey availability) decreases [11,22]. Therefore, it is important to understand the interactive role that corallivorous predators play in the survivorship of wild and restored coral populations.

**Fig 1:**
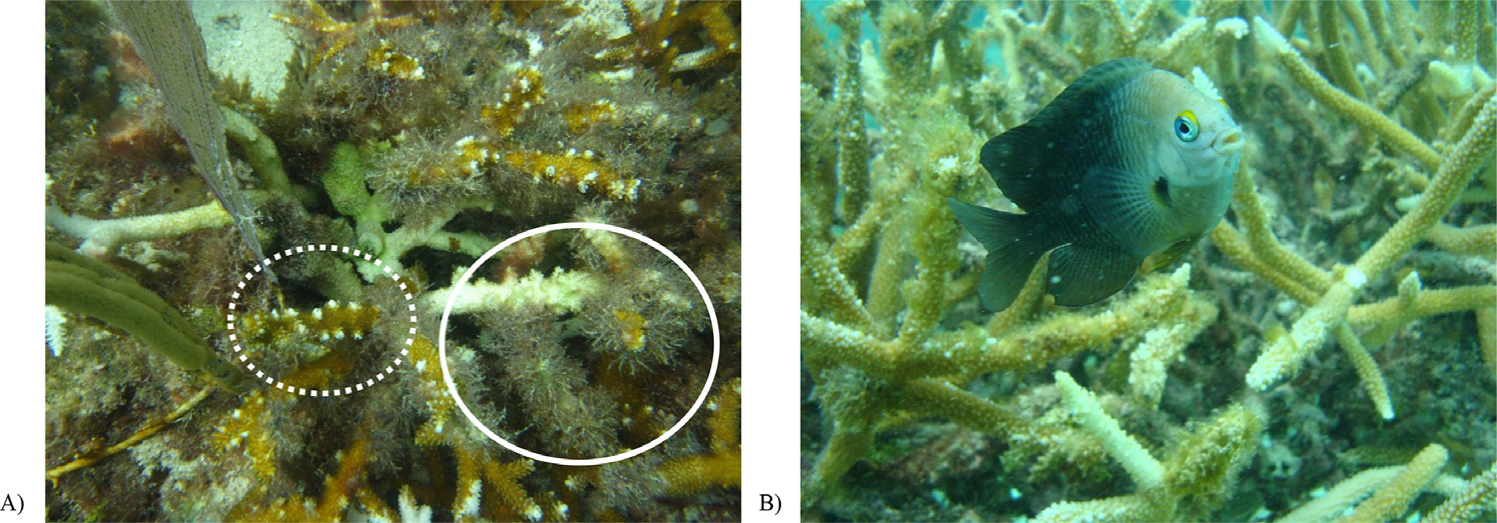
Colony of *Acropora cervicornis* with examples of (a) a damselfish algal lawn (solid circle) and chimneys formed by damselfish bites (dashed circle), and (b) a resident farming damselfish, *Stegastes planifrons*. Chimneys are formed as the coral attempts to recover tissue damaged due to repeated biting by damselfishes.

The cultivation of algal lawns by damselfishes can have detrimental effects on their coral hosts [7,23,24]. Farming damselfishes, particularly the threespot damselfish *Stegastes planifrons*, establish algal lawns by repeatedly biting live coral tissue and creating skeletal lesions that are quickly colonized by filamentous algae (Fig 1) [25–27]. Damselfishes aggressively defend their algal lawns thereby reducing grazing pressure of other herbivorous fish and invertebrates, allowing algae to grow within their territories and adjacent reef substrate [28,29]. Increased macroalgal abundance within territories establishes boundary competition between the surviving coral tissue and the algae, increases sedimentation as sediments are trapped by the algae, and reduces suitable substrate for the settlement of coral larvae [23,28,30,31]. Overgrowth by macroalgae, metabolic stress caused by algal competition, and energetic drain caused by lesion recovery can lead to total colony mortality [32,33]. Algal lawns also provide microhabitat for boring organisms thereby enhancing bioerosion of the coral framework and contributing to overall reef degradation [33–35]. Predation by territorial damselfishes may also create reservoirs of potential coral pathogens and increase the spread of disease [36].

In the Caribbean, damselfish territories can cover up to 80% of shallow reef habitat [37–39]. Threespot damselfish are known to prefer *A*. *cervicornis* as habitat, with threespot damselfish associated with *A*. *cervicornis* colonies 20 times more often than with any other microhabitat type [40]. Damselfishes may switch to less desirable coral hosts, like mounding *Orbicella* spp., when *Acropora* is not available [10]. With densities of damselfishes along the Florida Reef Tract (FRT) as high as 4.2 fish/m^2^ [41], the effects of damselfishes on benthic community dynamics may be substantial. The presence of damselfish lawns may also inhibit the settlement of *Acropora* larvae and the attachment of *Acropora* fragments [23,28,30], thereby reducing the overall success of *Acropora* beyond the direct damage inflicted on the adult host colony. As an example, damselfish gardening played an important role in the collapse of staghorn populations in Jamaica and dramatically slowed the recovery of *A*. *cervicornis* following Hurricane Allen [12]. Our present research on coral-damselfish interactions is especially relevant as this threatened keystone species is believed to be in a recovering trend after decades of decline [42].

Throughout the Caribbean, *in situ* coral nurseries are being used to propagate a sustainable source of *A*. *cervicornis* [3] and serve as genetic repositories [43]. Damselfish predation and algal farming on *A*. *cervicornis* colonies have been observed within coral nurseries and restoration sites [3,44,45]. In fact, predation by fishes and corallivorous gastropods can be a serious problem for newly transplanted corals [46]. Coral predators are found to redistribute to habitat with higher prey availability [47] and may have significant consequences on the survival of transplanted colonies. In Florida, the corallivorous snail *Coralliophila abbreviata* has shown a clear preference for transplanted *A*. *cervicornis* over other coral prey [19]. Therefore, it is important to document potential cascading effects as a result of these predator-prey interactions and fill knowledge gaps on how predation may affect both natural recovery and restoration success.

The three main objectives of this project were to: 1) evaluate the occupation dynamics of damselfishes on *A*. *cervicornis* colonies along the FRT, 2) determine the impacts of damselfish predation and algal lawn formation on the growth and survivorship of *A*. *cervicornis*, and 3) identify potential interactions between damselfishes and other *A*. *cervicornis* predators. The results of this study provide key insights into the dynamic nature of *Acropora*-damselfish interactions and how these relationships may affect the recovery of this threatened coral species.

## Methods

### Occupation Patterns and Impacts of Damselfishes on Wild *A*. *cervicornis*

**Fig 2:**
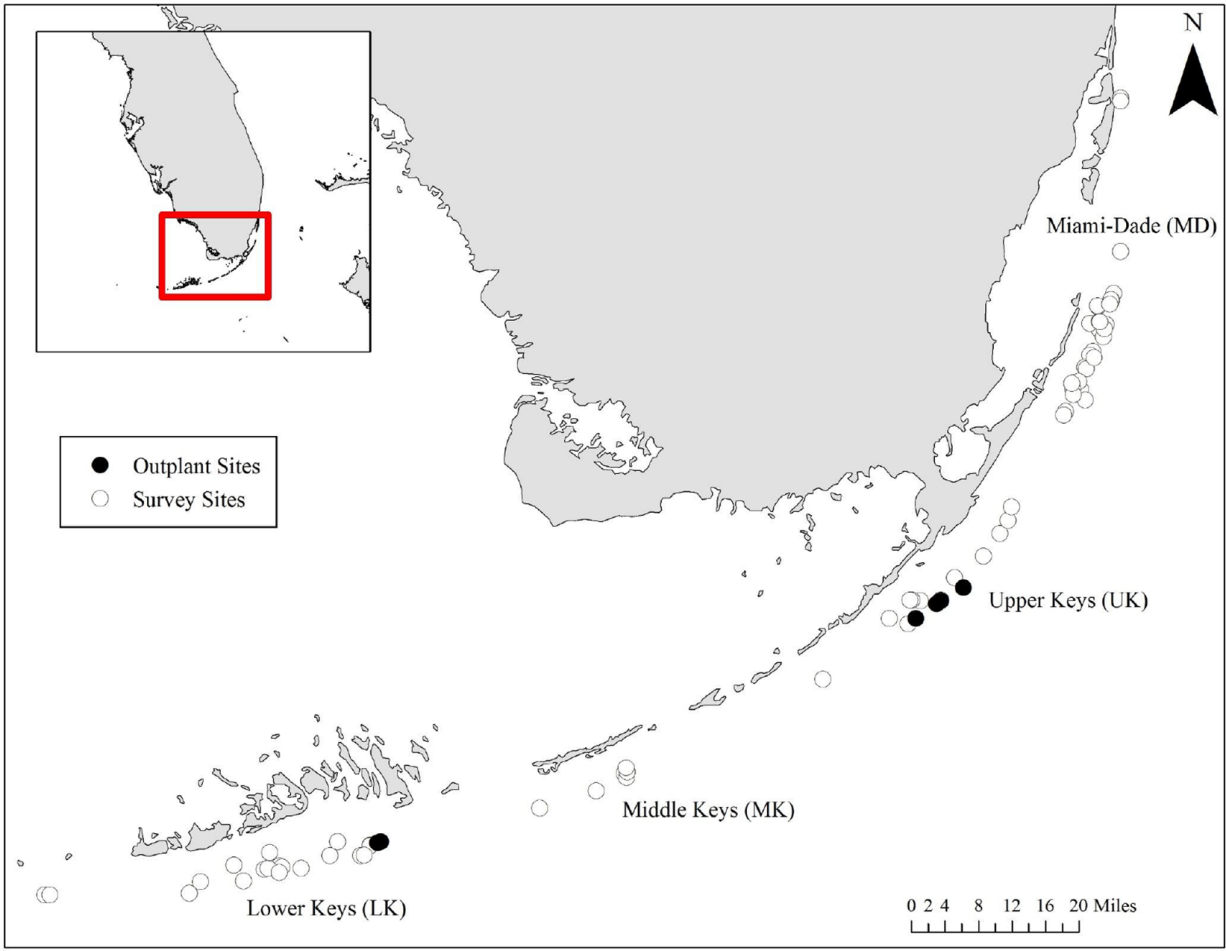
Location of sites along the Florida Reef Tract surveyed to investigate damselfish occupational dynamics and impacts. Open circles indicate sites where only wild staghorn colonies were observed. Black circles indicate sites where nursery-grown colonies were outplanted as part of restoration activities.

Roving-diver visual surveys (20–25 mins) were conducted in 2013 and 2014 to determine occupation patterns of damselfishes on colonies of *A*. *cervicornis* in four regions along the FRT (Miami-Dade County (MD), Upper Keys (UK), Middle Keys (MK), and Lower Keys (LK); Fig 2). The data collected were used to determine damselfish occupation patterns (i.e., the number, proportion, size, and complexity of staghorn colonies occupied by damselfishes) and occupation impacts (i.e., percent mortality due to damselfish gardening activities and territoriality). All *A*. *cervicornis* colonies (alive and dead) were measured for maximum diameter (cm), maximum height (cm), and number of branches (a proxy of colony complexity). Colony health and the severity of impacts were determined by estimating percent partial tissue mortality of each colony [48]. When partial tissue mortality was observed, the cause of mortality was assigned to the following categories/factors: 1) damselfish algal lawns, 2) damselfish bites/chimneys, 3) predation by corallivorous predators such as *Coralliophila* or *Hermodice*, 4) disease, and 5) other mortality (i.e., algal or sponge overgrowth). The occurrence of damselfish lawns was determined by the presence of a turf algal community overgrowth on the colony and/or damselfish bites/chimneys combined with the presence of a resident farming damselfish species, typically threespot (*S*. *planifrons*), dusky (*S*. *adustus*), or cocoa (*S*. *variabilis*) damselfishes (Fig 1). Prevalence (number of observations/total number of colonies) of each source of mortality was calculated. In addition to prevalence, the severity of impacts as the proportion of the coral colony affected by a mortality factor was recorded. Severity is calculated as the average impact for each factor for only those colonies where impacts were observed (thus excluding colonies were no impacts were observed).

### Progression of Damselfish Occupation Impacts on Wild *A*. *cervicornis*

Changes in size of established damselfish lawns within *A*. *cervicornis* colonies were documented quarterly for one year on wild colonies (n = 8) of similar mean maximum diameter (ANOVA; *p* = 0.171) and complexity (estimated as number of branches, ANOVA; *p* = 0.165) on three reefs within Miami-Dade County. Colonies (n = 8) from the same reefs and of similar size and complexity, but without lawns, were selected as controls. Colony health, size, and number of branches were assessed as described previously. Branch growth was measured between intervals by marking branches within each colony at a distance of 2 cm from the apical tip with a small cable tie [4,49].

### Occupation Rates and Impacts of Damselfishes on Outplanted *A*. *cervicornis*

Nursery-reared *A*. *cervicornis* colonies were outplanted in the Upper Keys (4 sites; Snapper Ledge, Pickles Reef, Conch Reef, Molasses Reef) and Lower Keys (2 sites; East Patch Looe Key, Looe Key ROA) to document damselfish occupation rates and subsequent occupation impacts (Fig 2). Nursery-reared *A*. *cervicornis* colonies (n = 10 for each size class) of three size classes (small: 10–15 cm; medium: 16–35 cm; and large: >36 cm maximum diameter) were secured to reef habitat at each site in a 5 x 6 m plot with masonry nails and cable ties [2]. Colonies were spaced 1 meter apart within each grid and monitored quarterly. In addition to these sparse grids, small thickets of corals were outplanted at East Patch Looe Key in the Lower Keys near existing threespot damselfish territories to promote and accelerate damselfish occupation by providing complex colony habitat. To create these thickets, 10 colonies (~20 cm maximum diameter) were outplanted (< 10 cm apart) into dense plots (n = 4). All outplants were monitored quarterly for one year to observe colonization of damselfishes to newly outplanted coral colonies and to document the rate and severity of algal lawn formation.

Research activities were conducted under permits from the United States Department of Interior National Park Service (BISC-2014-SCI-0018) and the Florida Keys National Marine Sanctuary (FKNMS-2013-090, FKNMS-2011-159-A1, FKNMS-2011-150-A1). Permission for research including endangered and/or protected species was provided by Special Activity Licenses from the Florida Fish and Wildlife Conservation Commission (SAL-13-1086-SCRP, SAL-14-1086-SCRP). Damselfishes were not handled as part of this study (all data were collected using visual surveys only) and IACUC approval was not required.

### Statistical Analyses

Our surveys and experiments included multiple colonies within sites. When the occupational impacts or sources of mortality were evaluated, “occupation” (presence or absence of damselfish) was considered as a fixed treatment, while “site” was treated as random nested factor within the ANOVA models. The same applies to multiple measurements (i.e., to document colony growth) that were obtained within the same colony. In such cases, both “site” and “colony” were treated as random factors nested within “occupation” in the statistical tests completed. The likelihood of damselfish occupation with respect to colony size (maximum diameter) and complexity (number of branches) was evaluated using logistic regression with damselfish presence-absence data. For this logistic regression, the variable “site” was nested within the predictor variables (size and complexity).

## Results

### Occupation Patterns and Impacts of Damselfishes on Wild *A*. *cervicornis*

A total of 921 *A*. *cervicornis* colonies from 78 sites were surveyed along the FRT (MD = 460; UK = 92; MK = 202; LK = 152). The damselfish species found associated with *A*. *cervicornis* were the non-farming species *Stegastes partitus* (bicolor), *S*. *leucostictus* (beaugregory), and *Microspathodon chrysurus* (yellowtail), as well as the farming species *S*. *planifrons* (threespot), *S*. *adustus* (dusky), and *S*. *variabilis* (cocoa) damselfishes. Damselfishes (all species included) were found on 30.9% of staghorn colonies surveyed.

**Fig 3:**
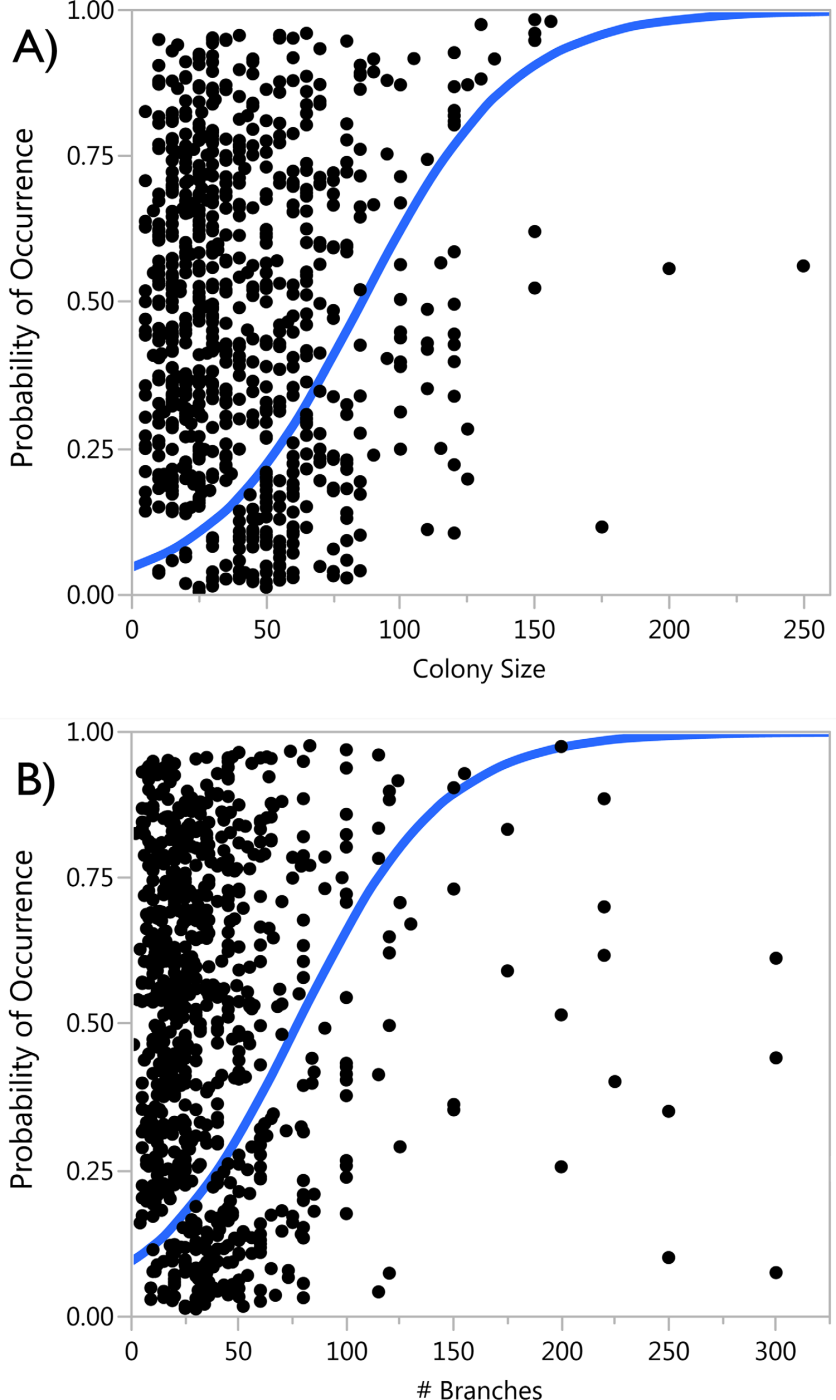
Probability of damselfish occupancy and formation of algal lawns based on *Acropora cervicornis* colony size and complexity (# of branches).

Mean diameter for all *A*. *cervicornis* colonies surveyed was 43.6 (SE = ± 1.3) cm with a mean height of 19.9 (0.4) cm and 35.6 (1.1) branches per colony. Most colonies were “healthy”, with a mean 71.5% (1.2) live tissue. The probability of damselfish occupancy (i.e., a colony being used as part of a damselfish territory) increased significantly with staghorn colony size (*p*<0.001) and number of branches (*p*<0.001; Fig 3) based on a logistic regression analysis. Thus, larger, more complex staghorn colonies are significantly more likely to be used as damselfish territories than smaller, less complex colonies.

**Fig 4:**
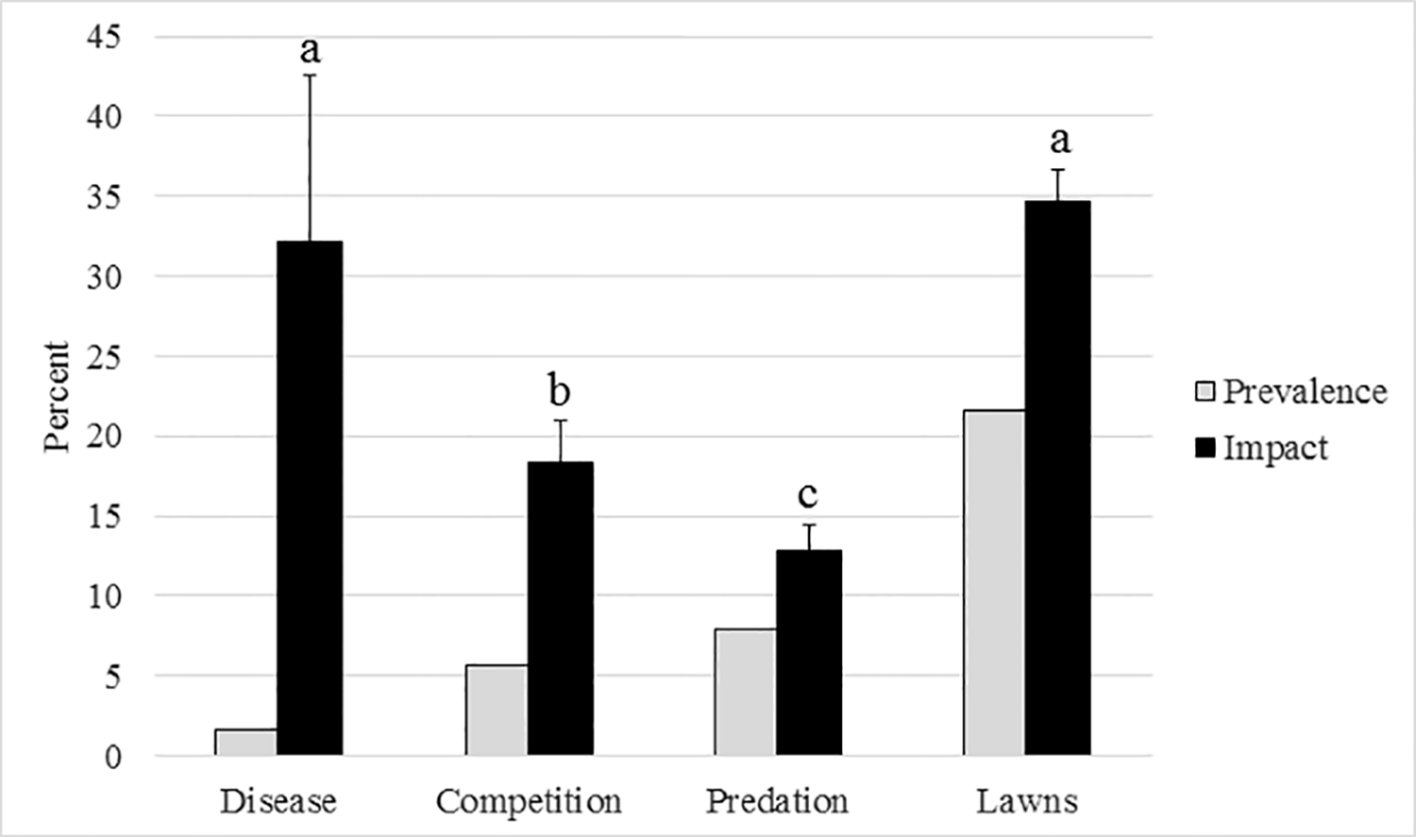
Prevalence of sources of coral mortality (gray bars) and severity of impacts expressed as the mean percentage of tissue mortality on *A*. *cervicornis* colonies (black bars ± SE). Letters reference statistical difference in the mean percentage of tissue mortality of each coral colony affected by mortality between sources (one-way ANOVA; *p*<0.001).

Low prevalence of predation by corallivores (*Coralliophila* and *Hermodice*) (7.9% of colonies) and overgrowth by algae or sponges (5.6% of colonies) was observed, with each factor causing relatively low tissue mortality (12.8 ± 1.6% and 18.3 ± 2.7% of the colony, respectively; Fig 4). Disease prevalence was low (1.6% of colonies), but, when present, disease caused high tissue mortality within colonies (32.1 ± 10.5% of tissue; Fig 4). Prevalence of damselfish lawns on *A*. *cervicornis* colonies (21.6% of colonies) was higher than the prevalence of any other mortality factor (i.e., predation, disease, algal or sponge overgrowth) and caused the highest tissue mortality (34.6 ± 2.1%; range 5–100%; ANOVA; *p*<0.001; Fig 4). Threespot damselfish were the most common species occupying colonies with algal lawns (42.4% of colonies), followed by cocoa damselfish (31.4%), bicolor damselfish (16.5%), and dusky damselfish (8.2%). Bicolor damselfishes were always found co-occupying colonies with other lawn-building species. The prevalence of corallivore predation was inversely related to the presence of a resident damselfish (Pearson’s *x* ^2^ = 7.61, df = 1, *p* = 0.006). The severity of corallivore predation was lower in colonies with algal lawns defended by damselfish (9.4 ± 1.0%) than in colonies without lawns (15.7 ± 2.7%; ANOVA; *p =* 0.055).

### Progression of Occupation Impacts of Damselfishes on Wild *A*. *cervicornis*

Over one year, the size of algal lawns within colonies increased by 5–10% in three occupied colonies. A threespot damselfish occupied one control colony (12.5% of unoccupied colonies), and formed a new lawn that caused 30% tissue mortality within six months. Damselfish occupation influenced coral growth, with mean annual growth rates for branches on colonies with lawns (15.4 ± 1.5 cm/yr) being significantly lower than on colonies without a lawn (29.6 ± 2.9 cm/yr; t-test, *p<*0.001).

### Occupation Rates and Impacts of Damselfishes on Outplanted *A*. *cervicornis*

Survival of nursery-reared *A*. *cervicornis* outplants was high at all sites (89.5 ± 2.4%) even after the severe 2014 summer bleaching event. No algal lawns were formed on outplanted corals (n = 60) in the Lower Keys, but five lawns were formed on outplants (n = 120) across three sites in the Upper Keys. In agreement with findings from surveys of wild colonies, lawn formation was related to colony size, and colonies occupied by damselfishes were significantly larger (t-test; *p* = 0.044) and more complex (t-test; *p* = 0.038) than outplants without lawn formation. Most lawns were formed within six months after outplanting and covered 14.0% (2.4) (range 10–20%) of the colony. Prevalence of corallivore predation was low on all outplants (14.1% of colonies), with feeding scars removing 11.7% (2.1) of tissue on affected colonies. As was the case for wild colonies, partial mortality due to *Coralliophila* and *Hermodice* was lower on outplanted colonies with a resident damselfish (5.0 ± 0.0%) than without (13.9 ± 2.3%) (t-test, *p* = 0.058). The large and complex coral thickets installed at East Patch Looe Key in the Lower Keys immediately attracted threespot damselfish, which began to utilize and defend the new territory even before divers had completed the installations. All thickets were colonized by threespot damselfish and algal lawns covered 20–45% of the colony within six months.

## Discussion

Damselfishes are important components of coral reefs, but their territorial behavior and gardening activities cause direct mortality to corals [7,32,33] and create negative cascading effects by reducing overall grazing on reef macroalgae [28,29]. In fact, the impacts of damselfish occupation have been recognized as a factor influencing coral reef community dynamics since the Pleistocene [10,24,50,51]. But, the steep decline in coral abundance, especially of those coral species that are commonly used by territorial damselfishes, established the present scenario in which damselfish impacts are now concentrated on a much lower number of coral prey/colonies, thus creating a significant threat to the persistence and recovery of depleted coral populations. Here, we show that territorial damselfishes are a significant source of mortality to recovering and restored populations of the threatened staghorn coral *Acropora cervicornis* along the Florida Reef Tract. Damselfish occupation and impacts are influenced by the size of the staghorn colonies, with larger, more complex colonies having a higher prevalence of damselfish occupation. Our transplant experiments clearly demonstrated that while staghorn colonies escape damselfish occupation at small sizes, colonies are quickly targeted by damselfishes after colonies reach diameters > 45 cm, and tissue mortality of up to 45% per colony can occur during just six months of damselfish occupation. Moreover, damselfish occupation results in significantly lower coral growth (branch linear extension) in host colonies. The concentration of impacts on remaining small staghorn populations is clearly a source of concern for the recovery of this threatened coral species and should be considered in restoration activities and management strategies.

Along the Florida Reef Tract, the > 900 wild *A*. *cervicornis* colonies surveyed were generally healthy, with the majority of colonies having > 90% live tissue. However, partial mortality caused by disease, predation, damselfish grazing, and other sources were documented on nearly half of all colonies. Similar to findings by Tunnicliffe [52], damselfish lawns were the most prevalent source of mortality to *A*. *cervicornis* and 34.6% of tissue mortality was caused directly by damselfish gardening activities. We documented significantly higher prevalence of damselfish lawns (21.6%) than surveys conducted along the FRT in 2008 (2.2%) [53], showing a concerning increase in the occurrence of damselfish occupation over a short time period. In addition to causing immediate tissue mortality, damselfish occupation negatively impacted coral growth. Growth rates of *A*. *cervicornis* were significantly lower in colonies with damselfish lawns. While the occupation rate of damselfishes to smaller outplanted colonies was low (5 out of 180 colonies over one year), occupation was clearly size-dependent with the probability of damselfish occupancy increasing as coral colony size and complexity increases. Thus, as colonies become larger and more complex (as shown by the higher occupation rates of the larger wild colonies and dense thicket outplants), damselfish occupation rates will also increase significantly. Considering that small *A*. *cervicornis* outplants (5 cm total linear extension) can reach colony sizes desirable as damselfish habitat (> 45 cm maximum diameter) within 1–2 years [4,54], reduced growth and productivity as a result of damselfish predation may prevent or delay colonies from reaching sexual maturity. Reduced growth of smaller colonies combined with mortality of larger, more mature (and fecund) *A*. *cervicornis* colonies may have a significant effect on population dynamics by potentially eliminating important genetic diversity and reducing the reproductive potential of depleted populations.

In this study, colonies with a resident damselfish exhibited lower prevalence of predation by *Coralliophila* or *Hermodice* and lower partial tissue mortality caused by predation. Coral predation has been linked to substantial and chronic mortality [55–58], prevention of recovery after acute disturbances such as storms [12], an increase in coral disease transmission [59,60], and impairment of propagation and outplanting phases of active *A*. *cervicornis* restoration projects [3]. Therefore, a reduction in *Coralliophila* and *Hermodice* predation as a result of the presence of a resident damselfish is an important finding of this study and may offset some of the negative impacts caused by damselfishes (i.e., the presence of a resident damselfish may reduce the spread of epizootic coral diseases by preventing *Coralliophila* grazing [59] although the potential of direct disease transmission by damselfish farming is still unknown [36]).

A potential mechanism for the reduction of damselfish impacts is to decrease the abundance of territorial damselfishes through the increased protection of damselfish fish predators with the establishment of no-take zones. The potential for predatory fish to control the abundance of damselfish, and thus mitigate damselfish impacts on restored corals populations, is in fact one of the reasons why restoration practitioners have proposed conducting coral restoration activities within Marine Protected Areas [2, 3]. Beneficial trophic cascades, if present, would clearly enhance the success of coral restoration efforts, but the reported relationship between the abundances of damselfish and their predators has been highly variable both spatially and temporally. DeMartini et al. [61] and Vermeij et al. [62] documented a negative correlation between the abundances of predatory fish and damselfishes, Catano et al. [63] showed damselfish abundance to be positively correlated with large predator biomass, Bohnsack [64] documented higher damselfish density in areas with high fishing pressure, and Mumby et al. [65] observed a reduction in damselfish density in overfished areas due to increases in mesopredators but did not find a change in density within protected areas. Finally other studies observed no change in damselfish density based on predator densities [10,66,67]. The variability in response of damselfish abundance to predator biomass may also be caused or mediated by other factors such as shelter availability [68,69] and recruitment [70]. While the abundance of predatory fish was not directly addressed within our study, focusing *Acropora* population enhancement within areas of potentially higher piscivore biomass, such as within no-take or protected areas, may provide additional protection against damselfish predation and should be considered a priority in the design of restoration strategies. In addition, it will be important to monitor fish abundance and community structure during restoration efforts to evaluate changes in trophic structure and predator-prey dynamics as *A*. *cervicornis* increases in abundance.

Gardening damselfishes were only found on *A*. *cervicornis* colonies with at least some live coral tissue, similar to findings by Precht et al. [10]. Few algal lawns were still found on colonies devoid of any live tissue and resident farming damselfishes were completely absent in those cases. Only bicolor damselfish, which do not farm lawns and utilize colonies for habitat only, were found on dead colonies [71]. This observation suggests that direct occupation impacts, as well as reduced growth of the occupied coral, often leads to complete host mortality and that damselfishes may vacate previously occupied colonies after colony demise. While the corallivorous snail *C*. *abbreviata* has been shown to stay on its prey until the tissue is completely consumed [19], damselfishes, who do not derive a primary dietary benefit from the coral tissue [24, 72], may be forced to abandon their territories if the cost of defending a lawn exceeds the benefit derived from it. The quality of a territory may decline as the lawns increase in size and the coral colony is unable to recover the tissue lost to damselfish bites due to reduced growth rates. Once algal lawns become too large, damselfishes may not be able to defend them against other herbivores or prevent overgrowth by larger, undesirable macroalgae. If the resident damselfish is unable to limit macroalgal growth within and around a coral colony, excess algal growth can eliminate space between coral branches, and reduce the amount of available shelter [63, 73–75]. At this point, damselfishes may move to establish territories on new, live colonies. Additional studies are needed to investigate such interactive effects between damselfish and their coral hosts to determine potential thresholds for algal farming activities.

The severity of coral predation can change radically depending on the size of the prey population via threshold effects and can strongly influence the potential recovery of depleted coral populations [76]. For example, in areas of high staghorn abundance, such as Jamaica or Panama prior to 1980 [25,52,77,78], damselfishes and other coral predators had limited effects on coral health, as their impacts were distributed across a large population. However, when Hurricane Allen caused dramatic declines in the abundance of *A*. *cervicornis* but only limited declines in coral predator density, the concentration of predator impacts onto small remnant coral populations effectively prevented coral recovery [11,12]. Even at low *A*. *cervicornis* densities, stable persistence of predators, such as damselfishes, *Coralliophila*, and *Hermodice*, is more likely if predators can occupy nearby, less preferred coral prey [10,12]. While *A*. *cervicornis* is the preferred prey for damselfishes and *Coralliophila* [19], and thus, is especially vulnerable to intense and focused predator activities, migration by damselfishes, especially threespots, onto less preferred coral hosts as the abundance of preferred prey declines has been observed [10]. Corals commonly targeted in the absence of *Acropora*, namely species of the boulder genus *Orbicella*, have also experienced drastic declines in abundance and are now listed as threatened under the US Endangered Species Act. In fact, Precht et al. [10] suggest planting faster-growing, nursery-reared *A*. *cervicornis* colonies in the vicinity of *Orbicella* colonies to reduce predation on the slower growing species while allowing damselfishes to return to their preferred prey/habitat. Although damselfish occupation often leads to complete host mortality of *A*. *cervicornis* and, therefore, would potentially sacrifice some staghorn corals, the ability to rapidly propagate and outplant large numbers of *Acropora* from coral nurseries may serve as a viable option to aid in the recovery of both genera.

This study documents dramatic differences in damselfish occupation rates of *A*. *cervicornis* based on colony size. As colonies become larger and more complex through high productivity and growth rates or through strategic thicket outplant designs employed during Caribbean restoration efforts, occupation rates also increase. Within very short time scales, outplanted *A*. *cervicornis* colonies can reach colony sizes targeted as damselfish habitat. Therefore, restoration planting strategies may be modified based on the abundance of coral predators at a given site. But, this may prove to be challenging as damselfish abundances are high throughout the FRT. Faced with the option of transplanting fewer but larger colonies or a greater number of smaller colonies, practitioners may decide to select the latter so that more colonies reach the size where they become desirable habitat to territorial damselfish at roughly the same time, thereby spreading the impacts of damselfishes among a larger number of hosts (assuming a set number of damselfishes are available to occupy these colonies). However, transplanting smaller colonies also has disadvantages as both survivorship and reproduction of corals are directly related to size [79].

The spatial arrangement of colonies within reefs can also influence damselfish impacts and need to be considered in outplanting design. Here, we showed that close spacing of outplants provides immediate habitat for damselfishes and has been shown to be detrimental to coral growth by Griffin et al. [54]. Thus, a wider spacing between colonies may be desired in reefs with higher damselfish abundance. Wider spacing of outplants is also supported by the work of Johnston and Miller [19] who showed that coral predator impacts are influenced by the composition of the coral neighborhood surrounding targeted species. The combination of high damselfish recruitment and reduced growth rates of closely-spaced colonies suggests that outplanting staghorn coral in dense aggregations or thickets may not be suitable for initial recovery of *A*. *cervicornis* under present conditions. The trade-offs outlined here should be addressed explicitly in future restoration efforts so that science-based recommendations for the active propagation and recovery of threatened coral species can be further refined.

This is the first study, to our knowledge, to evaluate the prevalence of resident damselfishes and the significant negative physiological effects of algal lawns on *A*. *cervicornis* along the FRT. Although damselfishes reduced predation severity by other corallivores, the fast rate of damselfish occupation and subsequent partial coral colony mortality by the creation of algal lawns raise concerns for the success of active restoration programs designed to enhance staghorn coral populations. However, most negative impacts of damselfishes identified in this study affected mainly large individual colonies and less than half of the *A*. *cervicornis* population along the FRT. The remaining wild staghorn population, along with the rapidly increasing restored population, continue to fulfill important functional roles on coral reefs such as providing essential habitat and refuge to other reef organisms. In addition, since damselfishes primarily favor larger colonies, the majority of outplanted colonies, as well as abundant smaller wild colonies, continue to have the opportunity to drastically increase total coral cover through rapid growth. While the effects of damselfish predation are, and will continue to be, pervasive, strategic outplanting designs may help overcome damselfish damage as coral abundance builds over time to the point where damselfish impacts are not overly concentrated.

## Supporting Information

S1 Data File Raw data collected during field surveys used within this study. (XLSX)

## References

[1] Hogarth WT. Endangered and threatened species: final listing determinations for the elkhorn coral and staghorn coral. Fed Reg. 2006 pp. 26,852–26,861.

[2] Johnson ME, Lustic C, Bartels E, Baums IB, Gilliam DS, Larson L, et al Caribbean *Acropora* restoration guide: Best practices for propagation and population enhancement The Nature Conservancy, Arlington, VA 2011.

[3] Young C, Schopmeyer SA, Lirman D. A review of reef restoration and coral propagation using the threatened genus *Acropora* in the Caribbean and Western Atlantic. Bull Mar Sci. 2012;88(4): 1075–1098.

[4] Lirman D, Schopmeyer SA, Galvan V, Drury C, Baker AC, et al Growth dynamics of the threatened Caribbean staghorn coral *Acropora cervicornis*: Influence of host genotype, symbiont identity, colony size, and environmental setting. PLoS ONE 2014;9(9): e107253 10.1371/journal.pone.0107253 25268812 PMC4182308

[5] Silbiger NJ, Guadayol O, Thomas FIM, Donahue MJ. Reefs shift from net accretion to net erosion along a natural environmental gradient. Mar Ecol Prog Ser. 2014;515: 33–44.

[6] Silverman J, Schneider K, Kline DI, Rivlin T, Rivlin A, Hamylton S, et al Community calcification in Lizard Island, Great Barrier Reef: A 33 year perspective. Geochim Cosmochim Acta. 2014;144: 72–81.

[7] Kaufman L. The threespot damselfish: effects on benthic biota of Caribbean coral reefs. Proc 3rd Int Coral Reef Symp, Miami, Florida. 1997: pp. 559–564.

[8] Williams AH. Ecology of threespot damselfish: Social organization, age structure, and population stability. J Exp Mar Biol Ecol. 1978;34: 197–213.

[9] Itzkowitz M. Spatial organization of the Jamaican damselfish community. J Exp Mar Biol Ecol. 1997;28: 217–241.

[10] Precht WF, Aronson RB, Moody RM, Kaufman L. Changing patterns of microhabitat utilization by the threespot damselfish, *Stegastes planifrons*, on Caribbean reefs. PloS One 2010;5(5): e10835 10.1371/journal.pone.0010835 20520809 PMC2877077

[11] Knowlton N, Lang JC, Rooney MC, Clifford P. Evidence for delayed mortality in hurricane-damaged Jamaican staghorn corals. Nature. 1981;294: 251–252.

[12] Knowlton N, Lang JC, Keller BD. Case study of natural population collapse: post-hurricane predation on Jamaican staghorn corals. Smithson Contrib Mar Sci. 1990;31: 1–30.

[13] Powles H, Bradford MJ, Bradford R, Doubleday W, Innes S, Levings CD. Assessing and protecting endangered marine species. ICES Journal of Marine Science: Journal du Conseil. 2000;57(3): 669–676.

[14] Roby DD, Lyons DE, Craig DP, Collis K, Visser GH. Quantifying the effect of predators on endangered species using a bioenergetics approach: Caspian terns and juvenile salmonids in the Columbia River estuary. Can J Zool. 2003;81(2): 250–265.

[15] Engeman RM, Constantin B, Gruver KS, Rossi C. Managing predators to protect endangered species and promote their successful reproduction In: Columbus AM, Kuznetsov L, editors. Endangered Species: New Research. Hauppauge, NY: Nova Science Publishers; 2009 pp. 171–187.

[16] Baum JK, Worm B. Cascading top‐down effects of changing oceanic predator abundances. J Anim Ecol. 2009;78(4): 699–714. 10.1111/j.1365-2656.2009.01531.x 19298616

[17] D'Antonio C, Meyerson LA. Exotic plant species as problems and solutions in ecological restoration: a synthesis. Restor Ecol. 2002;10(4): 703–713.

[18] Ruiz‐Jaen MC, Aide TM. Restoration success: How is it being measured? Restor Ecol. 2005;13(3): 569–577.

[19] Johnston L, Miller M. Negative indirect effects of neighbors on imperiled scleractinian corals. Coral Reefs. 2014;33(4): 1047–1056.

[20] Moran PJ. The *Acanthaster* phenomenon. Oceanogr. Mar. Biol. Annu. Rev. 1986;24: 379–480.

[21] Hughes T, Connell J. Multiple stressors on coral reefs: A long‐term perspective. Limnol Oceanogr. 1999;44(3;2): 932–940.

[22] Rotjan RD, Lewis SM. Impact of coral predators on tropical reefs. Mar Ecol Prog Ser. 2008;367: 73–91.

[23] Potts D. Suppression of coral populations by filamentous algae within damselfish territories. J Exp Mar Biol Ecol. 1977;28: 207–216.

[24] Lobel PS. Herbivory by damselfishes and their role in coral reef community ecology. Bull Mar Sci. 1980;30: 273–289.

[25] Robertson DR, Hoffman SG, Sheldon JM. Availability of space for the territorial Caribbean damselfish *Eupomacentrus planifrons* . Ecology. 1981;62: 1162–1169.

[26] Sammarco P, Williams AH. Damselfish territoriality: Influence on Diadema distribution and implications for coral community structure. Mar Ecol Prog Ser. 1982;8: 53–59.

[27] Hixon MA, Brostoff WN. Succession and herbivory: Effects of differential fish grazing on Hawaiian coral-reef algae. Ecol Monogr. 1996;61(1): 67–90.

[28] Vine P. Effects of algal grazing and aggressive behaviour of the fishes *Pomacentrus lividus* and *Acanthurus sohal* on coral-reef ecology. Mar Biol. 1974;24: 131–136.

[29] Belk MS. Habitat partitioning in two tropical reef fishes, *Pomacentrus lividus* and *P*. *albofasciatus* . Copeia 1975;4: 603–607.

[30] Hixon MA. Effects of reef fishes on corals and algae In: Birkeland C, editor. Life and death of coral reefs. New York: Chapman and Hall; 1997 pp. 230–248.

[31] Gordon TAC, Cowburn B, Sluka RD. Defended territories of an aggressive damselfish contain lower juvenile coral density than adjacent non-defended areas on Kenyan lagoon patch reefs. Coral Reefs. 2015;34: 13–16.

[32] Myrberg AA Jr, Thresher RE. Interspecific aggression and its relevance to the concept of territoriality in reef fishes. Am Zool. 1974;14: 81–96.

[33] Sammarco PW, Carleton J, Risk MJ. Effects of grazing and damselfish territoriality on bioerosion of dead corals: Direct effects. J Exp Mar Biol Ecol. 1986;98: 1–19.

[34] Risk MJ, Sammarco PW. Bioerosion of corals and the influence of damselfish territoriality: A preliminary study. Oecologia. 1982;52: 376–380. 28310398 10.1007/BF00367962

[35] Hutchings P. Biological destruction of coral reefs. Coral Reefs 1986;4: 239–252.

[36] Casey JM, Ainsworth TD, Choat JH, Connolly SR. Farming behaviour of reef fishes increases the prevalence of coral disease associated microbes and black band disease. Proc. R. Soc. B; 2014;281: 20141032 10.1098/rspb.2014.1032 24966320 PMC4083805

[37] Williams AH. The effects of Hurricane Allen on back reef populations of Discovery Bay, Jamaica. J Exp Mar Biol Ecol. 1984;75: 233–243.

[38] Axline-Minotti BA. The role of threespot damselfish (*Stegastes planifrons*) as a keystone species in a Bahamian patch reef. M. Sc. Thesis, Ohio University. 2003.

[39] Grober-Dunsmore R, Bonito V, Frazer TK. Potential inhibitors to recovery of *Acropora palmata* populations in St. John, US Virgin Islands. Mar Ecol Prog Ser. 2006;321: 123–132.

[40] Clarke R. Habitat distribution and species diversity of Chaetodontid and Pomacentrid fishes near Bimini, Bahamas. Mar Biol. 1977;40: 277–289.

[41] Wilkes AA, Cook MM, DiGirolamo AL, Eme J, Grim JM, Hohmann BC, et al A comparison of damselfish densities on live staghorn coral (*Acropora cervicornis*) and coral rubble in Dry Tortugas National Park. Southeastern Naturalist. 2008;7(3): 483–492.

[42] Gardner TA, Côté IM, Gill JA, Grant A, Watkinson AR. Long-term region-wide declines in Caribbean corals. Science. 2003;301(5635): 958–960. 12869698 10.1126/science.1086050

[43] Schopmeyer SA, Lirman D, Bartels E, Byrne J, Gilliam DS, Hunt J, et al *In situ* coral nurseries serve as genetic repositories for coral reef restoration after an extreme cold‐water event. Restor Ecol. 2012;20(6): 696–703.

[44] Hernandez-Delgado EA, Rosado Matias BJ, Sabat AM. Restauracion del habitat esencial de peces juveniles mediante le replantacion de corales fragmentados en la Reserva Pesquera Marina del Canal de Luis Pena, Culebra. Mem. XXIV Simp Rec Nat. 2001: 77–97.

[45] Miller MW, Wilborn R, Higgins J. Aquarius coral restoration/resilience experiment (ACRRE): Interim Report, NOAA Report # PRBD-09/10-6. 2010. pp. 1–17.

[46] Omori M. Degradation and restoration of coral reefs: Experience in Okinawa, Japan. Mar Biol Res. 2011;7(1): 3–12.

[47] Wellenreuther M, Connell SD. Response of predators to prey abundance: separating the effects of prey density and patch size. J Exp Mar Biol Ecol. 2002;273(1): 61–71.

[48] Lirman D, Formel N, Schopmeyer SA, Ault JS, Smith SG, Gilliam D, et al Percent recent mortality (PRM) of stony corals as an ecological indicator of coral reef condition. Ecol Indic. 2014;44: 120–127.

[49] Shinn EA. Coral reef recovery in Florida and the Persian Gulf. Environ Geol. 1976;1(4): 241–254.

[50] Kaufman L. There was biological disturbance on Pleistocene coral reefs. Paleobiology 1981;7(4): 527–532.

[51] Knowlton N. Thresholds and multiple stable states in coral reef community dynamics. Am Zool. 1992;32(6): 674–682.

[52] Tunnicliffe V. Caribbean staghorn coral populations: pre-hurricane Allen conditions in Discovery Bay, Jamaica. Bull Mar Sci. 1983;33: 132–151.

[53] Miller SL, Chiappone M, Rutten LM, Swanson DW. Population status of *Acropora* corals in the Florida Keys. Proc 11th Inter Coral Reef Symp. Ft. Lauderdale, Florida. 2008.

[54] Griffin JN, Schrack EC, Lewis K, Baums IB, Soomdat N, Silliman BR. Density‐dependent effects on initial growth of a branching coral under restoration. Restor Ecol. 2015;23(3): 197–200.

[55] Brawley SH, Adey WH. *Coralliophila abbreviata*: a significant corallivore. Bull Mar Sci. 1982;32: 595–599.

[56] Hayes JA. Distribution, movement and impact of the corallivorous gastropod *Coralliophila abbreviata* (Lamarck) on a Panamanian patch reef. J Exp Mar Biol Ecol. 1990;142(1): 25–42.

[57] Grober-Dunsmore R, Bonito V, Frazer TK. Potential inhibitors to recovery of *Acropora palmata* populations in St. John, US Virgin Islands. Mar Ecol Prog Ser. 2006;321: 123–132.

[58] Williams D, Miller M. Attributing mortality among drivers of population decline in *Acropora palmata* in the Florida Keys (USA). Coral Reefs. 2012;31(2): 369–382.

[59] Williams DE, Miller MW. Coral disease outbreak: Pattern, prevalence and transmission in *Acropora cervicornis* . Mar Ecol Prog Ser. 2005;301: 119–128.

[60] Sutherland KP, Shaban S, Joyner JL, Porter JW, Lipp EK. Human pathogen shown to cause disease in the threatened eklhorn coral *Acropora palmata* . PLoS ONE 2011;6(8): e23468 10.1371/journal.pone.0023468 21858132 PMC3157384

[61] DeMartini EE, Friedlander AM, Sandin SA, Sala E. Differences in fish-assemblage structure between fished and unfished atolls in the Northern Line Islands, central Pacific. Mar Ecol Prog Ser. 2008;365: 199–215.

[62] Vermeij MJA, DeBey H, Grimsditch G, Brown J, Obura D, DeLeon R, et al Negative effects of gardening damselfish *Stegastes planifrons* on coral health depend on predator abundance. Mar Ecol Prog Ser. 2015; 528: 289–296.

[63] Catano L, Gunn B, Kelley M, Burkepile D. Predation risk, resource quality, and reef structural complexity shape territoriality in a coral reef herbivore. PLoS ONE 2015;10(2): e0118764 10.3354/meps10921 25714431 PMC4340949

[64] Bohnsack JA. Effects of piscivorous predator removal on coral reef fish community structure In: Caillet GM Simenstad CA, editors. Fish food habits studies. Seattle: Washington SeaGrant Publication, University of Washington; 1982 pp. 258–267.

[65] Mumby PJ, Steneck RS, Edwards AJ, Ferrari R, Coleman, Harborne AR, et al Fishing down a Caribbean food web relaxes trophic cascades. Mar Ecol Prog Ser. 2012; 445, 13–24.

[66] Williams AH. An analysis of competitive interactions in a patch back-reef environment. Ecology. 1981;62(4): 1107–1120.

[67] Beukera JS, Jones GP. Habitat complexity modifies the impact of piscivores on a coral reef fish population. Oecologia. 1997;114: 50–59. 10.1007/s004420050419 28307557

[68] Holbrook SJ, Schmitt RJ. Competition for shelter space causes density-dependent predation mortality in damselfishes. Ecology 2002;83(10): 2855–2868.

[69] Feary DA, Almany GR, McCormick MI, Jones GP. Habitat choice, recruitment and the response of coral reef fishes to coral degradation. Oecologia 2007;153(3): 727–737. 17566781 10.1007/s00442-007-0773-4

[70] Sponaugle S, Cowen RK. Larval supply and patterns of recruitment for two Caribbean reef fishes *Stegastes partitus* and *Acanthurus bahianus* . Mar Freshwater Res. 1996;47(2): 433–447.

[71] Nemeth RS. The effect of natural variation in substrate architecture on the survival of juvenile bicolor damselfish. Environ Biol Fishes. 1998;53: 129–141.

[72] Jones GP, Santana L, McCook LJ, McCormick MI. Resource use and impact of three herbivorous damselfishes on coral reef communities. Mar Ecol Prog Ser. 2006;328: 215–224.

[73] Munday PL. Fitness consequences of habitat use and competition among coral-dwelling fishes. Oecologia. 2001;128: 585–593 28547404 10.1007/s004420100690

[74] Klumpp D, Polunin N. Partitioning among grazers of food resources within damselfish territories on a coral reef. J Exp Mar Biol Ecol. 1989;125(2): 145–169.

[75] Foster SA. Size-dependent territory defense by a damselfish. Oecologia. 1985;67: 499–505. 28311034 10.1007/BF00790020

[76] May RM. Thresholds and breakpoints in ecosystems with a multiplicity of stable states. Nature. 1977;269: 471–477.

[77] Goreau TF. The ecology of Jamaican coral reefs. Species composition and zonation. Ecology. 1959;40(1): 67–90.

[78] Kinzie RA. Coral Reef Project (III) Papers in Memory of Dr. Thomas F. Goreau, 5: The zonation of West Indian gorgonians. Bull Mar Sci. 1973;23: 93–155.73.

[79] Hughes T, Jackson J. Population dynamics and life histories of foliaceous corals. Ecol Monogr. 1985;55(2): 142–166.

